# Treatise on Diagnosis and Semeiology

**Published:** 1838-07

**Authors:** 


### Art. X.

1. *Traité de Diagnostic et de Séméiologie.* Par P. A. Piorry, m.d. &c. *Treatise on Diagnosis and Semeiology.* By P. A. Piorry, m.d. &c.—*Paris*, 1837-8. Three Vols. 8vo. pp. 611, 548, 651.

2. *Grundriss der Speciellen Semiotik.* Nach den Quellen bearbeitet, von Dr. H. E. Suckow, Kreisphysicus in Jauer.—*Jena*, 1838. *Outlines of Special Semeiology.* By Dr. H. E. Suckow.—*Jena*, 1838. 4to. pp. 296.

The first of the above works, the only one deserving special notice in this place, professes to be practical, and to be founded upon the physiological or physico-chemical laws of the economy. It is appropriately dedicated to MM. Bally and Orfila: to the former, for having devoted the experience acquired at the bedside to the improvement of therapeutics, as well as because a great number of the facts collected were observed in his service at the Hôtel Dieu; and to the latter, for his application of physical and physiological laws to the history of the effects of poisons and medicines: and, consistently with such a dedication, the author claims for his work no other merit than that it consists of a collection of facts verified by himself.

The commendation, however, that he disclaims for himself, he loses no opportunity of invoking upon his *organo-pathological nomenclature*, to which he devotes an introduction of twenty-seven pages. The following table of the new terms, anglicised, will give our readers a better idea of this nomenclature than any lengthened description of it.

| Names of certain Organs. | | | | | |
|---|---|---|---|---|---|
| English. | Greek. | Changed in Composition. | Termination. | Prefix. | Epithets. |
| | | | -pathic, *affection* | | |
| | | | -hemia, *sanguineous congestion* | hyper-, *increased degree* | Tubular |
| Stomach | Gaster | Gastro- | -itis, *inflammation* | hypo-, *deficiency* | Cancerous |
| Intestine | Enteron | Entero- | -trophy, *volume* | poly-, *multitude* | Hydatic |
| Lung | Pneumon | Pneumo- | -algia, *pain* | a-, *want or absence* | Epidemic |
| Womb | Metron | Metro- | -nervy, *nervous action* | dys-, *difficult* | Endemic |
| Brain | Encephalon | Encephalo- | -rhagia, *flow of blood* | hydro-, *watery* | Intermittent |
| Bone | Osteon | Osteo- | -rhœa, *colourless discharge* | aero-, *gaseous* | Typhoid |
| Joint | Arthron | Arthro- | -artia, *contraction* | toxico-, *poisonous* | Choleric |
| | | | -extasia, *distension* | pyo-, *purulent* | Saturnine |

Thus, an apoplexy is a *Hemo-encephalorrhagia;* a congestion of the brain from plethora is an *Encephalohemia-polyhyperhemica ;* and an emphysema is an *Aero-dermo-ectasia!*

Now, before we use our privilege as critics with regard to this nomenclature, let us see on what grounds the author recommends it. The reasons adduced are, first, that "in medicine a nomenclature is indispensable;" though no cause is alleged why it is so, except we take the following, "that it is absurd to be continually employing terms that have no correspondence with the things implied by them." But, granting for a moment the sufficiency of these reasons, are the terms used altogether free from ambiguity? Certainly not; for M. Piorry himself confesses that most of them are arbitrary, and that some have been perverted from their primary and general signification to a secondary and restricted sense: thus, -rhagia and -rhœa, in the original Greek, express different degrees of the same thing, (viz. an impetuous or gentle current,) yet one is used to describe a flow of blood, and the other of white or colourless discharges. Besides making these admissions, M. P., like certain lawyers who have a weak cause to defend, brings forward objections for the purpose of showing how easily he can overthrow them. The first is, the length of the terms. The example, Hyper-spleno-trophy; to which he replies, It is much longer to say, an increase of volume of the spleen. Hence, it conduces to rapidity and conciseness of style to substitute a word for an expression. We will reserve what we have to say with regard to the above example, and pass on to the next objection.

2d. The terms are harsh and inharmonious. Example, Cardiarctia. The reply is, first, These words have been admitted into science; and it is better to admit them, though they are inharmonious, yet are understood, than to coin others more agreeable, but whose meaning nobody can find out without hunting in a dictionary. In this reply, M. P. has supplied his opponents with a most powerful argument against his nomenclature; for we are quite certain that no person, however adorned with classical acquirements, can make out such terms, even with the aid of all the Scapulas and Hedericks in the Bodleian, unless he is acquainted with the organo-pathological nomenclature, from the author's works themselves; particularly when one-half of a term may have a Greek and the other a Latin derivation, as in the example before us. It is, however, clear that M. P. rather prides himself upon this hybrid creation of his organo-pathological imagination; for he asserts that Cardiarctia, in the difficulty of its pronunciation, expresses the obstacle to the flow of blood through the contracted heart! We think that he might have gone even further in his onomatopœa; for we cannot utter the word without eliciting a *bruit de scie* from our lips.

3d. It is assuming the province of scientific bodies. Reply, Great bodies move slowly, and the nomenclature is not the original work of one person, but a collection of terms, most of which have already been employed.

4. It creates new words. Reply, No: it is a combination of terms already in use.

5th. What is the use of changing the terms? Why not retain the adopted language, when everybody understands its meaning? Reply, The adopted terms are not understood: for an apoplexy with one is a congestion, with another a hemorrhage. Fever is an entity that everybody speaks of, but nobody understands. The only way to extricate one's self from the labyrinth of skin-diseases is to name each organically; i. e. from the tissue affected. The new nomenclature remedies these abuses: it does not give a definite name to the disease, but it clearly expresses the state of the organs, in the opinion of the person who employs it; but, if another be of a different opinion, he is enabled by it to express himself in adequate terms. Thus, if there be a question respecting a gastric pain, one may call it a Gastritis, another a Gastralgia, and a third a Gastrohemia; and, if the case be obscure, they may all concur in terming it a Gastropathy.

Here, again, we think M. Piorry unfortunate in his choice of arguments; for, if the affection be thus obscure that it cannot be decided whether it be an inflammation, a neuralgia, or a congestion, it appears to us that the original expression, "gastric pain," is quite as clear as gastropathy: and, with regard to diseases of the skin, as we shall see hereafter, the application of the new nomenclature is a signal failure; for it not only admits of the old arrangements into papular, pustular, &c., but, by classing them all as Dermites, or inflammations of the skin, considered as a whole, it imposes a character upon them that is far from correct in all instances; for it is evident that the same tissues cannot be affected in a pustular or papular disease as in the squamous or the exanthemata.

As to the ambiguity of the term apoplexy, no one, we conceive, in using it, does more than apply a name highly expressive of the character of a set of symptoms, that are better understood by this appellation than if he were to refer them to their organic cause, whether congestion, rupture of a blood-vessel, or a softening of the brain.

The great fear of our author seems to be, lest, by giving determinate names to diseases, medical men should fall into the error of expecting to discover specific modes of treatment for them, and thus neglect the consideration of the pathological states on which they depend. On this objection to the old terms, with the present thirst for knowledge and spirit of enquiry that distinguishes medical men, we do not think it necessary to bestow any consideration.

We shall not dwell upon two other rather weak objections to the nomenclature,—viz. that it is difficult to acquire, and is not likely to become popular; because, if it be worth anything, it is worth learning; and, though it may not become familiar, men of science will take pains to understand it. But there are still two more, and in our eyes very formidable, objections, which M. P. has failed in overcoming, and which, in reading recent French medical works, are a great source of annoyance; viz. that every scribbler, now-a-days, takes the liberty of enveloping his crude conceptions in a shroud of long-winded terms, without, in some cases, giving any explanation of what he intends by them; pretty much in the same barbarous style that a corncutter terms himself a Chiropodist and Corn-exantlator; and that another Worthy of the same stamp, terms a washball by the sonorous name of Rypophagon. The other objection is still more serious; namely, that in many cases the new nomenclature imperfectly describes the disease, or cannot describe it at all in the present state of our knowledge, or rather ignorance, of the subject. To this M. P. replies, that we may either use the old terms, or may each of us apply such a name as we think most closely coincides with our views on this subject. In the former alternative we fully agree with the author, but the latter is open to all the objections expressed above.

After stating our objections to M. Piorry's nomenclature at such length, our conclusion may appear strange, but we give it with perfect good faith; viz. that we consider it, with one or two exceptions, etymologically well-formed for its purpose, and in many cases very expressive: moreover, we shall not hesitate to avail ourselves of it whenever we find that the old and adopted terms are inadequate to express our meaning so fully and so concisely. But, to repeat our objections briefly, we protest against the use of definite names for undetermined diseases, and we dislike that established and well-understood terms should be changed merely because they are not of themselves a definition of the thing they represent. As an instance of the former impropriety, we cite Hypersplenotrophy, as indicative of the state of the spleen in ague; which is not an hypertrophy, but only an enlargement or distension: and of the latter, the disuse of the word rheumatism, because it implies an erroneous view of the nature of the disease.

After the introductory chapter upon his Organopathological Nomenclature, our author enters upon his work with two short sections upon Diagnosis and Semeiology in general, and upon the mode of examining a patient; from which we shall only extract one most important yet rather neglected hint,—viz. that a judicious method of examination gives the patient the highest confidence in his physician: to which we would beg leave to add, that, if the patient do not object to them, the physician cannot be too minute in his enquiries, if he wishes to attain the same end. He then enters fully into the causes, symptoms, and physical signs of disease, in reference to the diagnosis, which he further considers specially and comparatively. He next points out the necessity of keeping in view the effects of treatment as a source of correct diagnosis; defends the moderns from the charge of neglect of prognosis, which he asserts they draw more justly from the exact state of the affected organ than from physiological or pathological symptoms, as the ancients did; and concludes this part of his work with a table of the questions to be put, and of the routine to be followed, in examining a patient. With respect to the imputation of neglecting prognosis, we must say that we think our Gallic brethren are rather open to it; and we found our opinion not only on our own observation in French hospitals, but upon the numerous cases published in the different journals, of patients bled, day after day, till within a few hours of their death; nay, even up to the last moment.

The first volume is devoted to the diseases of the organs of Circulation and Respiration; the second, to those of the Digestive and Generative Organs; and the third, to those of the Skin, the Nervous System, the Muscles, and the Bones. But, before entering upon the special diagnosis of the diseases of any organ, M. Piorry gives an account, not only of every mode by which information of the state of the organ may be acquired, but also of the diagnostic value of the result obtained; so that the special diagnosis is little more than a mere enumeration of certain symptoms and signs, whose relation to the disease has been already proved and established. This plan has the advantage of placing before the student at once every thing that can elucidate the different diseases of each organ; but, if he has already formed his diagnosis, it very much increases the difficulty and trouble of ascertaining its correctness, particularly if many of the symptoms be of a sympathetic character; for these are described with the affections of the organ they implicate, not in connexion with that part with which they sympathise: thus, for the state of the eye in epilepsy, the reader is referred to the section on the eye, to that on the nerves, and to the special diagnosis of the disease itself.

M. Piorry devotes 120 pages to the exploration of the heart and to the special diagnosis of its diseases, in which we have found many points of interest, particularly in the results of examination by percussion.

He attaches but little importance to the motions of the epigastrium, as indicative of adhesion of the pericardium, or of fluid in its cavity; and he omits entirely the rubbing sensation occasionally imparted to the hand under the former circumstance. He claims for percussion not only the power of determining the limits of the heart, but of indicating the position and size of the right and left cavities, and, "approximatively," the thickness of their walls.

M. P. has proved by experiment that the volume of the heart may be greatly increased by congestion of its substance when its cavities are perfectly empty; and it is to the removal of this state by bleeding that we must refer the great diminution in its size frequently consequent upon that operation. In the normal state, the heart is in contact with the ribs over a space of twenty to twenty-four lines in extent; and it usually reaches, covered by the lung, an inch and a half or two inches further to the left, as may be detected by powerful percussion: its normal transverse diameter is therefore from three and a half to four inches. From an examination of 177 cases of various diseases, it appears that those attended with disordered respiration were more or less conducive to an augmentation of the heart's size. The vertical dimension of the heart is generally a little less than the transverse; and the distance of the heart below the clavicle is usually from three to three and a half inches, but this space is often diminished one or two inches by hypertrophy or by pressure of the diaphragm. In the above 177 cases, the dimensions, during life, occasionally exceeded those found after death from the cause above stated (the congestion of the heart), as well as from its distance from the parietes and the convexity of the chest; all of which have the same tendency to increase the extent of the dull sound. At the right of the normal dull space, there is dulness without resistance to a slight extent, corresponding to the extension of the right cavities beyond the left on that side; while to the left we have both dulness and resistance combined, arising from the greater thickness of the left ventricle. In hydropericardium with much fluid, there is a dull sound over a pyramidal space, extending from the upper part of the sternum to the region of the heart. When the fluid is in a less quantity, it is detectible by dulness along the right or left side of the upper part of the sternum, according to the side upon which the patient is lying. It is distinguished from dilatation of the right cavities by extending round the great vessels to either side the sternum; whereas, in dilatation, the dulness is chiefly to the sight of the sternum, or under the right side of that bone.

Although the rules laid down by Laennec and succeeding authors are generally correct with regard to the loudness of the sounds being proportionate to the thinness of the walls of the heart, yet M. P. has frequently found the parietes thickened when the sound was clear, and *vice versâ;* and the tone will frequently vary in the same persons, if subject to palpitations. He attaches but little importance, therefore, to the differences in clearness of tone and to the extent over which the sounds of the heart are audible, the latter being modified by so many circumstances; but he attributes the former to the quantity of blood contained, the sound being clearest when the heart is most empty, yet is contracting with energy, as in palpitations: these points, however, he thinks, require further investigation. The impulse offers the same uncertainties; and is rather an indication of the force of the blow than of the thickness of the walls. In the aged females at Salpetrière very little impulse has been observed, though the organ was often much thickened; and in nervous subjects with small hearts the impulse is frequently very strong. A good illustration is taken of the state of action of the heart in such cases, from the struggles of an hysterical female, in whom there is most powerful muscular energy under excitement combined with general debility. He does not attempt any explanation of the double, and even triple impulse described by M. Bouillaud.

In some cases he has observed only twenty-eight or thirty pulsations in a minute, and in one instance only seventeen. He quotes M. Bouillaud's observation of double and triple sounds being confined to persons whose hearts have their orifices contracted; and states that the distance of the sounds of the heart is no proof of hydropericardium, as it is produced by the overlapping of the lung also, and is noticed in cases of extreme hypertrophy.

Upon the value of the bellows-sounds as a sign of diseased valves, M. Piorry is quite at issue with M. Bouillaud, having frequently heard it when no disease existed, and *vice versâ.* He quotes a letter from M. Dechambre respecting eighty-four patients at Salpetrière: of fifty-eight without disease, the bellows-sound was heard in three; of twenty-six cases of indurations or cartilaginous ossifications of the valves or orifices, the sound was heard occasionally or permanently in only seven; and in some of the cases, where there was no sound, the contraction was greater than where it was heard. Of thirteen other cases of contracted orifices, (seven extensively so, six in a less degree,) the bellows-sound was heard in six of the former and only two of the latter. Various other observations of his own and of M. Louis are cited in support of this fact, which our own experience enables us to confirm. The account of the other abnormal sounds presents no novelties to those who have attended to the writings of Corrigan, Spittal, Stokes, &c.; to whom it is pleasing to see that our author, though a Frenchman, will allow some little merit.

In summing up the diagnostic value of the sounds, he gives a preference to the results of percussion, and offers a judicious caution not to insist too much upon the stethoscopic indications, to the exclusion of the more practical physiological symptoms, by which at last our treatment must be guided.*

* M. P. says, with great naïveté, that, when we have established the position and dimensions of the heart by percussion, we must be very careful to mark them out on the body of the patient with lunar caustic!!

From the special diagnosis of diseases of the heart we have extracted the following particulars:

*Congestion of the Heart:* Disturbed circulation and respiration, with extensive dulness on percussion, arising suddenly, and quickly removed by bleeding.

*Carditis:* When a sudden acute pain, particularly if rheumatism be present, is felt exactly where the heart strikes the ribs, and nowhere else, we may suspect carditis. Abscess, softening, &c. cannot be ascertained during life.

*Pericarditis:* In moderate effusion, the dulness is found to be vertical rather than transverse over the heart.

*Atrophy:* No symptoms are given, but they are the reverse of hypertrophy. To avoid error, it is necessary to percuss with force, and to recollect that, where the liver does not advance to the left side, the heart sinks low in the epigastrium.

*Dilatation* of the right auricle produces a dull sound at the upper part of the sternum, and is distinguished from hydropericardium by not extending to the left side.

In the exploration of the aorta, M. Piorry places great reliance upon percussion, which, in case of an aneurism, will not only give a dull sound over the part where the tumour is in contact with the walls of the chest, but also, if pretty forcible, will indicate the whole circumference of the aneurism, whether it be of the ascending or descending aorta: only, in the latter case, percussion must be applied on each side of the spine. This examination requires the greatest caution, lest too great violence should rupture the tumour; and hence, in these cases, the pleximeter is indispensable. In proof of the great accuracy of this mode of exploration, M. P. was enabled, by the difference of the sounds elicited, to define the extent of an aneurism, and of the right auricle in contact with it. In an appendix he states that, by pressing his fingers down beneath the sternum, while the neck was bent to relax the mastoids, he was enabled to feel a dilatation of the aorta, in a case in which he had suspected its existence from the dulness observed over a circular circumscribed portion of the sternum.

After some minute but useful directions respecting the method of feeling the pulse, M. Piorry proceeds to the indications derivable from its various modifications. He does not attempt (perhaps judiciously) to account physiologically for all the variations in its frequency, but contents himself with an enumeration of their occasional causes. We must not omit, however, that he adheres firmly to Broussais's opinion that the quickness of the pulse in fever is produced by the communication of irritation to the ganglionic system, and so to the heart, from the inflamed bowels. Upon the whole, he places no reliance upon the rapidity of the pulse indicating any organic affection, irrespective of other morbid phenomena; but he considers a state of fever to exist when the pulse constantly exceeds its normal number by eight or ten beats. His observations upon the other characters of the pulse are exceedingly just, and well worthy the attention of the student; but he has omitted to mark sufficiently strongly the hemorrhagic pulse, perhaps the most difficult of all to appreciate, and yet the most important to distinguish in practice. In order to prove how little the pulse is to be relied on in forming a diagnosis, he gives a table of 100 cases, taken at random, in which the pulse was accurately noted, and shows that almost every variety of pulse was present under similar circumstances of disease: still, however, a general character was observable in the majority of cases of the same affection. He also makes some most useful remarks upon the effects of change of position of the limbs upon the pulse, and on their diagnostic value; and he questions the accuracy of Dr. Corrigan's conclusion that, when an increase of the force of the pulse at the wrist is induced by elevating the arms, it is a pathognomonic sign of insufficiency of the aortic valves.

M. Piorry has never observed a sense of pain and heat along the vessel in arteritis.

The necessity of attending to the state of the veins in certain obscure affections of the abdominal viscera is inculcated, (§ 656,) particularly where obliteration of the cava is suspected; but we do not find any hint given of a phenomenon we have occasionally noticed in obstruction of the internal veins,—viz. that the blood will pursue a retrograde course in the cutaneous vessels, and thus restore the circulation.

The first of the following observations (§ 711) is highly important; the other (§ 712) is likely to mislead. It is a mistake (says M. P.) to suppose that a lymphatic temperament is one in which the lymphatic vessels are more particularly active: on the contrary, they are weaker than they ought to be, like varicose veins, and, like them, their distension depends in great measure upon debility of the heart and circulating organs. It does not follow, he adds, that, because a child has swelled glands, it is of a scrofulous habit; we often find an obvious cause in a carious tooth or the irritation of teething: nevertheless, the engorgement, once established, is not only continued, but even extends to other parts of the system, in virtue of the law of extension laid down by Bichât. Here we differ from M. P.; for we do not believe that the glandular affection will extend itself and become general, except in persons of a scrofulous constitution.

The following is interesting, as bearing upon the origin of *Tubercle.* In fourteen cases M. P. has observed "greyish granulations" in the buffy coat of the blood. Their colour was well marked at their centre, but it passed insensibly at the edges into that of the surrounding mass; their size varied from that of a poppy-seed to a hemp-seed; and in many cases they could be separated by the point of the scalpel from their matrix, when they were found to be of a dark-grey colour, and soft: hard enough, however, to admit of division by the scalpel. They had no true pus at their centre. Where they did not admit of separation from the buff, they were easily rendered visible at its surface, by tearing it across, perticularly if held up against the light. In one mass of buff seen by Andral, there were above fifteen granulations; and, when examined by M. Donné by a microscope, they were found to be minute clots of solid matter contained in the cells of the buffy coat. In all these cases there were decided chronic inflammations, more particularly of the lungs. In one instance, where the patient was seventy-one, there was purulent urine, followed by pneumonia, obliteration of the brachial arteries, and softening of the brain. These fourteen were all the cases out of 10,000 where the blood was observed with care, and in all the fourteen pus was detected either in the sputa or urine, during life or after death, in some of the organs; and hence we must consider this circumstance as a sign of pus in the blood.

In this section upon the Blood there is a great want of proper arrangement of the subjects; there are frequent repetitions; and the relations of the chemical states of the blood to disease are far too lightly touched upon.

We set our faces most strongly against the doctrine (§ 779, &c.) that the state of the blood indicated by the buffy coat is a constant attendant upon inflammation, or that it is always indicative of its presence. We need not refer to the appearance of the blood in pregnancy and bronchitis, in illustration of this error; for, in another paragraph (§ 780), he admits that, of seventy-six venesections performed in twenty-nine cases of inflammatory disease (not bronchitis), the blood of sixty only exhibited the buffy coat. To this state of the blood M. P. has applied (improperly, in our opinion, and inconsistently with his own nomenclature,) the name of Hemite, or Hemitis: and we object to this term, because, as he and other pathologists have adopted the termination *-itis* as expressing inflammation of the organ or tissue to which it is attached, others, as well as ourselves, may be led into the error of supposing that Hemitis means that the blood itself is inflamed, which is not the case.

Our readers may form some opinion of the minute attention that M. Piorry pays to his subject, from his devoting twenty pages to the exploration of the nasal fossæ; from the whole of which we are sorry that we can find nothing to transcribe that is worth their attention. In the thirty-eight pages taken up by the larynx and trachea, also, there is little to detain the reviewer or man of experience, though the student will find some useful hints, drawn from various sources, upon the examination of these organs.

Under the head *Palpitation in the Exploration of Bronchial Disease,* M. Piorry remarks upon the burning heat of the skin in certain cases of bronchitis; so that it would appear that this symptom, recently enlarged upon by Dr. Addison, is not confined to inflammation of the substance of the lung. Indeed, although M. P. asserts that the temperature of the body in diseases of the lungs is remarkably disturbed, and that, in almost all cases where the heat is very great there is an increase of action, either general or partial, of the lungs, yet he nowhere gives the slightest hint of its being a pathognomonic sign of pneumonia; which, were it so, such an accurate observer would hardly have failed to notice. The voice, cough, and rhonchus or wheezing, afford considerable assistance in forming a diagnosis of bronchial and pulmonary disease; and, although we cannot altogether agree with M. P. in ascribing a necessary mutual dependence of these sounds, yet we think there is a sufficiently intimate relation between them, particularly when modified by disease of the bronchi or lungs, to deserve more attention than it has hitherto met with, either from writers or lecturers on these subjects.

M. Piorry is very unwilling to refer the absence of the respiratory murmur to any other than organic causes; and hence he thinks the existence of such a disease as nervous asthma very doubtful; and ascribes the physical signs of emphysema of the lung, as given by Laennec, to distension, not rupture, of the vesicles, caused by the obstruction to the return of the air along the minuter ramifications of the bronchi, in consequence of the mucous secretion always present in chronic bronchitis, of which emphysema is a result. In another place, in reference to this subject, he claims precedence of M. Reynaud for the discovery, due in fact to Reisseisen, that, from the want of communication between the bronchi, the function of that portion of the lung to which an obliterated or obstructed bronchus leads must be lost to the economy.

The propositions laid down in the article upon *Percussion*, in the section devoted to the *Exploration of the Lungs*, are highly valuable; and, to use our author's words, " inasmuch as they establish the presence of a solid, liquid, or gaseous body in the lungs, admit of great exactitude;" and we perfectly agree with him, that, to practise percussion as it ought to be done, an accurate acquaintance is required with the anatomy of the thoracic organs, and of their condition and mutual relations both in health and disease. We cannot, however, discuss this subject without remarking that, though in a work on diagnosis it is essential that the student should be put into possession of every fact that may be useful to him in the investigation of disease, yet we think it doubtful whether such extreme nicety of touch is attainable by the majority as is said to be necessary, in some instances quoted, for the ascertaining the exact state of the organ examined. We allude to the assertion in § 1286, that the want of elasticity, as well as the dulness of sound, on percussion, will establish accurately the fact of a *deeply-seated* portion of the lung being indurated. In the correctness of the latter sign we agree, because the sound emitted is derived from all the organs to which the shock of percussion is communicated; but the elasticity depends almost entirely upon the resilience of the ribs and their covering, aided no doubt by the lungs beneath, which, we conceive, would afford an equally elastic substratum, whether their centres were indurated or not. Indeed, in his 18th Proposition, he states that circumscribed indurations of the lung, when small, do not give a different sound from the healthy lung: *a fortiori*, then, a deeply-seated one would be still less detectible by its want of elasticity. Another proof of the extreme delicacy of discrimination said to be attainable by means of percussion is M. P.'s assertion, that Laennec and Andral are both wrong in supposing that crepitation is audible in pneumonia before the resonance of the chest is affected; whereas, he maintains that the resonance is first diminished, which is certainly more conformable to what is observed in external inflammations,—viz. that congestion and engorgement of the tissue, pulmonary or other, precedes effusion, whether into the vesicles and minute bronchi, or into the cellular tissue of external parts. He attributes the presumed error of these excellent pathologists and auscultators to their using their fingers instead of the pleximeter.

The section upon *Auscultation of the Lungs* is meager, and proves that he places far more reliance upon the results of percussion than of auscultation. Great attention is paid to the consideration of the sputa; but the test for pus, proposed by Dr. Young, by refracted light, is omitted.

Upon the whole, after all we have constantly heard of the skill of the French physicians in diagnosis, we must say that we have risen rather disappointed from the perusal of this first part of M. Piorry's work.

Except in the application of percussion,—in which we are bound to acknowledge him to be *facilè princeps*, and which we regret to observe is much neglected in this country, though far more easily learnt to a certain degree, and therefore in one respect more useful than auscultation, —we remark an almost entire unacquaintance with the important recent discoveries in the diagnosis of chest disease that have been published in this country, of all which Dr. Stokes has so carefully availed himself, and to which he has himself so largely contributed in his excellent work on Diseases of the Chest.

In the forty-seven pages devoted to the *Exploration of the Mouth*, there are one or two subjects considered that are deserving attention, although we do not receive our author's conclusions as by any means established. One is, the inflammation of the parotid observed during the course and convalescence of fever, which M. P. says is not a critical phenomenon, but arises from the retention of the saliva in the duct in consequence of the obstruction of its orifice by the mucus of the mouth, which, dried by the passage of the breath, not only plugs up the mouth of the canal, but produces its ulceration, and the consequent inflammation of its whole extent to the gland. In proof, he alleges that, when the orifice is set free, there is first a discharge of pure pus, followed by pus and saliva mixed, and then by a return of the natural secretion, and by the subsidence of the glandular enlargement. We are aware that this fact admits of a different explanation, but we think it a point worth attention on practical grounds. The other subject is the condition of the tongue in various diseases. In 100 cases of typhus with enteritis, the state of the tongue was very variable, although the gastro-enteritic symptoms continued throughout the course of the disease. Bloodletting produced paleness of the organ, and the redness of the point was caused solely by the compression of the sides when the tongue was protruded. It was not red in sixty cases of organic lesions of the stomach and bowels. In 100 cases of pneumonia, or general hyperhæmic states not complicated with gastric symptoms, there was vivid redness of the tongue, followed by paleness after bleeding. From these observations the conclusion is, that the redness of the tongue indicates the state of hæmatosis and of the circulation, rather than the condition of the alimentary canal.

The dryness of the tongue is referred, in all cases, to the evaporation of its secretion by the air respired; and this opinion is attempted to be supported by many arguments, which, however, have failed in convincing us of its truth.* The fur on the tongue and teeth is regarded as merely dried saliva; and all the varieties of colour presented by the secretions of the mouth may, it is asserted, be produced by drying this fluid, or the tartar of the teeth, at a temperature of 100°, more or less rapidly, after it has been for some time exposed to the air. The loss of speech, in severe cases of typhus, is said to be owing merely to the dryness and stiffness of the tongue, and may be instantly relieved by moistening the mouth of the patient. The observations upon the acidity of the saliva do not altogether confirm those of M. Donné: "In the present state of our knowledge, (says M. P.) we cannot ascertain the condition

* We think our author's salivary organs must be peculiarly active, if they have never failed in their duty under the desiccating influence of a hot day, salt food, a slight excess in wine, an extra cigar, or even of a strong moral impression.

of the stomach or other organs from the acid, neutral, or alkaline state of the saliva; and further researches into this subject are necessary before any decided opinion can be formed." (§ 1484.) The mercurial fætor, according to M. Piorry, is nothing more than the odour arising from the fur and tartar that the increased secretion of saliva deposits upon the lining membrane of the mouth. This is our own opinion, except that we do not refer the secretion to the saliva alone, but think that it is formed also by the mucous membrane.*

We were not aware of the fact stated by M. P., that redness of the fauces is an ordinary symptom of plethora, especially in women at the menstrual period, when it assumes the form of congestion of the tonsils, but is immediately relieved by venesection. This symptom, if a true one, may be useful as a guide to treatment in other affections arising at such periods, when we are often in doubt as to the propriety of depleting measures.

The whole article upon the *Exploration of the Stomach, and the special Diagnosis of its Diseases*, though containing little or nothing but what an attentive consideration would suggest, is exceedingly useful, as bringing together, in a well-arranged form, all the various affections of the organ, and the different symptoms and physical signs that, if not infallible guides, are sufficiently certain to obviate any very egregious errors in the diagnosis.

The following account of the diagnostic powers of percussion, when applied to this organ, however, rather savours of the marvellous.—M. Fabre ascertained by this means that the stomach was contained in a scrotal hernia.—Percussion will determine whether the stomach be full or empty, and consequently its size and extent; the period during which the food remains in it; its contents if liquid, and the extent of tumours developed in it or in connexion with it. M. Piorry even looks forward to percussion being the guide to the operation of gastrotomy, if ever it be performed.

We think the following remark upon *Hemorrhage from the Bowels* (Enterorrhagy) worth noticing:

> "When, in a case of Enterorrhagy, we find that the iliac regions give a dull and fluctuating sound on percussion, we are not to conclude that the blood may not proceed from the rectum. In more than ten cases I have discovered, in piles, or in ulcerations of the rectum above the sphincter, the source of hemorrhagies that have been followed by accumulations of blood in the colon and cæcum. In such cases, therefore, we must examine the rectum most carefully." (§ 1757.)

Taking a review of the *Diseases of the Alimentary Canal*, we cannot forbear to notice that, while describing a set of organs that have the most intimate connexion with, and are acknowledged by all physiologists to be most particularly under the influence of, the nervous system, M. P. should have scarcely hinted at any other than organic and local causes for all the host of symptoms enumerated.

* We are presented, in § 1491, with a lamentable picture (and we fear it is by no means a solitary instance) of the evils resulting from trusting to theoretic notions instead of observation of disease. It seems that certain persons (disciples of Broussais, we imagine,) attach great importance to a fætor of the breath, termed "gastric," as indicating disease of the stomach; and a lady, who presented this symptom from a quantity of carious teeth, was actually nearly starved to death, in consequence, by her physician, who kept her without food for thirty days!

Under *Exploration of the Liver*, we are presented with an interesting account of the dimensions of that organ in health and disease, as indicated by percussion, and confirmed in many cases by examination after death: but we should have been much better pleased if M. Piorry could have given us some directions for detecting the various chronic diseases, unattended with much change of volume, to which the liver is so liable, and which are far more interesting to the practical physician than the temporary increase of bulk induced by congestion or inflammation, that admits of detection also by other means besides percussion. Not the slightest reference is made to the valuable researches of Mr. Kiernan, throughout the whole chapter; but, as we conceive it will be more interesting to our readers that we should cull M. P.'s excellencies than expose his defects, we will proceed to give the results of his mensuration of the liver in health and disease.

| No. of Cases. | | Towards Axilla. | Towards the Nipple. | In the Epigastrium. | To left of Median Line. |
|---|---|---|---|---|---|
| | *In Health* . | 4 inches | 3 inches | $2\frac{1}{2}$ inches | 2 inches |
| | *In Disease*— | | | | |
| 55 | Typhus . . | $5\frac{1}{2}$ | $4\frac{1}{2}$ | 3 — | 2 |
| 19 | Bronchitis . | $5\frac{7}{12}$ | 5 | 3 — | $1\frac{1}{8}$ |
| 24 | Phthisis . . | $5\frac{1}{3}$ | $4\frac{1}{3}$ | $2\frac{2}{3}$ | $1\frac{1}{2}$ |
| 8 | Rheumatism . | $5\frac{7}{12}$ + | $4\frac{7}{12}$ + | 3 | $2\frac{1}{2}$ |
| 24 | Hepatitis . | 7 — | $5\frac{1}{2}$ | $4\frac{1}{6}$ | $3\frac{1}{3}$ |
| 82 | Ague . . . | $5\frac{1}{2}$ — | 4 + | $2\frac{3}{4}$ + | 11 lines |
| 22 | Pneumonia . | $5\frac{1}{4}$ | $4\frac{1}{3}$ | 3 | $1\frac{1}{2}$ |
| 9 | Heart Disease | $6\frac{2}{3}$ | $5\frac{1}{3}$ | $3\frac{1}{6}$ | $1\frac{1}{2}$ |

From the above table we may infer, 1st, that, in hepatitis, the vertical diameter of the liver is two inches above the normal dimension, and that the transverse diameter is also increased. 2d. That, in disease of the heart, attended with dyspnœa, the liver is augmented in size. 3d. In bronchitis, frequently attended with obliteration of the bronchi and severe dyspnœa, the dimensions are less than in the former diseases. 4th. In pneumonia, the left side is larger than in bronchitis. 5th. That there is hepatic hypertrophy in rheumatism also. 6th. That, in ague, the liver is less enlarged than in rheumatism, bronchitis, or heart disease.

The presence of an hydatid cyst in the liver is detectible by percussion, to which it returns a sound that is compared to that of a repeater-watch, or a metal-spring cushion when struck with the finger, together with a sensation of striking upon a gelatinous matter. Of thirty-eight cases of hepatic disease, only four presented the symptom of pain in the right shoulder.

The spleen can only be examined by the touch when it is so much enlarged as to extend beyond the edge of the ribs; so that, of 500 cases in which it was hypertrophied, in only one-fifth was M. P. able to detect its extension into the hypochondriac region. Neither is the absolute size of the organ to be ascertained by this means; for, in some subjects, it forms a considerable projection beyond the ribs when only slightly enlarged, in consequence of not rising high under the diaphragm in the normal state. In other cases, on the contrary, it hardly advances beyond the bounds of the chest when its diameter vertically is $6\frac{1}{2}$ inches. In this uncertainty of the normal extent of the spleen, percussion offers itself as the only mode of examination that admits of accurate results; and M. P. somewhat exultingly points to his discoveries respecting the state of this organ in ague, as a touchstone of the superiority of percussion by the pleximeter over that by the fingers, which fails in giving such accurate results. As his discoveries on this point of pathology are quite original, we think the following sketch of his mode of proceeding to detect the state of the spleen will be acceptable to our readers.

First, the extent of the left lung is traced downwards in a direct line from the axilla, till powerful percussion indicates, by a dull sound in place of the clear pulmonary resonance, the presence of the spleen deeply seated beneath the ribs; next, by the same means, is found the point where the spleen is in contact with the abdominal parietes; lastly, these two points being determined, and also the limits of the heart, lung, liver, and kidney, it becomes easy to circumscribe the extent of the spleen in the other directions, except backwards towards the spine: but the difficulty of tracing the organ in that direction may be considerably lessened if the distension of the stomach and colon by solid, fluid, or gaseous matter is removed previous to the examination.

The healthy proportions of the spleen are as follows: In its vertical diameter it is from 3½ inches to 3¾ inches, and, in the transverse, 3 inches. It is situated some inches to the left of the median line, and rarely ever in health projects beyond the edge of the ribs. Its increase of size in disease is usually proportionate in all its dimensions: hence, in the subjoined account, its vertical diameter alone is given. In fifteen cases of pneumonia, it was 4 inches; in thirty-eight of phthisis, 3½ inches; in thirty-three of gastro-enteritis, 4½ inches: several of these cases were attended with rigors, and the spleen was contracted by the use of quinine. In twenty-three cases of hepatitis, it was 3¾ inches; in 130 agues, 5¾ inches. In most of these cases, its breadth and thickness were equal to the height. These results, and the observation of above 500 cases of ague, have convinced M. Piorry that the spleen is invariably enlarged (hypertrophied) or painful in ague; but that, in other diseases, a great increase in its size is only observed where periodic febrile attacks have occurred; and this has been confirmed by various observers both in France and Algiers.

The correct admeasurement of the spleen has established the following facts with regard to the use of quinine in agues: 1st. It reduces the volume of the spleen not only when it is greatly, but even when moderately, enlarged. 2d. The diminution bears some proportion to the quantity of the medicine taken; and this effect may be produced in half an hour; the urine also becoming very bitter in a very short time. We have ourselves seen, in the wards of M. Bally, a case in which the spleen, projecting two inches beyond the ribs, became hardly perceptible the next day after a large dose of quinine had been taken. The effect produced by quinine upon tertians and quartans is proportionate to the reduction of the spleen; so that the disease is cured simultaneously with restoration of this viscus to its healthy dimensions. On the other hand, though the symptoms be arrested, they will be liable to recur as long as the spleen exceeds its proper size. As the spleen attains its greatest enlargement at an early period of the disease, it proves that it is not the paroxysms that produce the hypertrophy, but rather the enlarged organ that maintains the disease. In the opinion of the author and M. Bally, no other remedy is so certain or energetic in agues as quinine; and its powers are equally remarkable in the ascites that results from long-continued disease of the spleen.

We had marked many passages for extraction connected with this novel and most interesting subject; but, as we find that we should exceed our limits were we to give them all in full, and we have already quoted sufficient to point out the author's views, we beg to refer our readers to the chapter itself upon the Spleen, which will amply repay them for its perusal.

M. Piorry has but little to add to the observations of Drs. Bright, Osborne, &c. upon *Albuminous Urine:* he has however observed a puriform deposit in the urine in several cases where the buffy coat of the blood was granulated, and where pus existed in the substance of the lungs. Another case of pneumonia rapidly recovered after a similar deposition had occurred in the urine, that continued some days. In phthisical subjects, also, who have exhibited no other sign of renal affection, albuminous urine has been noticed. From these and other facts, he concludes that a transient appearance of albumen in the urine does not alone warrant the admission of organic disease of the kidneys.

The third volume opens with the *Exploration and Diagnosis of Diseases of the Skin.* This account extends to 150 pages, of which ninety-five are occupied by the former subject, comprising the indications to be derived from the inspection and examination of the skin, with regard to its colour, temperature, consistence, &c., as well as from the consideration of its physiological relations, and the influence it exerts and receives by its sympathy with other organs in a state of disease. With his usual industry, M. Piorry has examined the temperature of above ninety persons in health and disease, and the following is a summary of the results: Of twenty-one healthy individuals, from seventeen to eighty years of age, the temperature of the axilla and groin (and it was always alike in both) varied from 97°.25 to 106.°25 F.; the difference between the eldest and youngest varied from 2°.25 to 4°.50 F. In six cases of fever (typhoid), the temperature was from 108°.50 to 117°.50 F.; in one of these the blood was 113° F. In fifteen cases of phthisis, from 97°.25 to 114°.12, the hands being always hotter than in health. In eight of pneumonia, from 99°.5 to 113°: in one instance, the hand was 115°.25, and the blood only 113°, (unless this is a misprint.) In fourteen cases of inflammation of the uterùs and vagina, the axilla was from 101°.75 to 110°.75, but the vagina from 104° to 117°.50.

In the above abstract we have been careful in converting the degrees of Reaumur, given in the original, into the equivalents of Fahrenheit, as we were struck with the remarkable difference of several of them from the statements of preceding authorities. Thus, in the observations of Dr. Currie, the temperature of fever is stated, at most, at 107°; we ourselves have seen it at 108°; it is stated by Donné at between 103° and 104°.* The only observations with which we are acquainted, that at all approach the high standard recorded by M. Piorry, are given by Dr. Granville, in a paper of Sir E. Home's, in the Philosophical Trans-

* British and Foreign Med. Review, Vol. I. p. 247.

actions for 1825; in which the temperature of the uterus, during and after parturition, is mentioned as being, in different cases, 100°, 105°, 108°, 110°, 118°, 120°. We call the attention of our readers to this subject, as we confess we have great doubts of the accuracy of the observations which give so elevated a degree of temperature. If incorrect, the inaccuracy may be readily explained by the well-known differences that exist among thermometers; a difference which is inevitable, unless every thermometer is individually regulated by a standard, which we fear is far from being the general practice of thermometer-makers.

Among the various morbid sensations of the skin, symptomatic of other diseases, he mentions one that has been generally overlooked, except in cases of neuralgic disorder; viz. an excessive tenderness of the skin, increased by the slightest touch, together with headach and muscular pains throughout the side opposite to the affected part of the head. This, in many cases, is indicative of a commencing softening of the brain; and the correctness of the diagnosis has been verified by dissection.

In speaking of the relation of the skin and the primæ viæ, M. P. states that his experience is contrary to the prevailing opinion (in France) of the dependence of erysipelas upon disorder of the digestive organs. Of 100 cases, only three were traceable to such an origin, and almost all the rest originated in some external cause.

M. Piorry opens the subject of the *Special Diagnosis of Skin Diseases* by finding fault with all the classifications hitherto used for their divisions and subdivisions, the neglect of the anatomy and physiology of the skin, and disregard of the coexistent states of the viscera, &c. of the system generally; for all which evils he proposes his own arrangement and nomenclature as a panacea eminently practical. We have attentively perused this section, and we consider it the feeblest part of his whole work; for he has not only added nothing to our knowledge, but, by the introduction of his nomenclature, he has left confusion worse confounded. Indeed, it is evident to us that his acquaintance with cutaneous disease is very limited, and derived rather from books than practice. We therefore pass on to the subjects of the *Eye* and the *Ear*, under which we find little to detain us, except a mode of ascertaining the soundness or imperfection of the drum of the ear, that we think worth mentioning: it consists merely in propelling the air along the Eustachian tube into the drum, by forcible expiration while the nostrils and lips are closed, when, if the membrana tympani be imperfect, the air will pass through it with a rushing or squeaking sound, audible to a bystander, and very perceptible by the patient.

About ninety pages (a space commensurate with the importance of the subject) are occupied with the *Exploration of the Brain and its Membranes*, which M. P. has treated in a very systematic manner, avoiding the fault that we have noticed in other parts of his work, of entering fully into the description of the diseases in order to explain the origin of certain of their symptoms. Almost the whole of this section merits close attention from the student, though there is little that is novel in the observations. Under *Mensuration of the Skull*, he remarks the occasional concurrence of atrophy of a portion of the brain with a want of symmetry of the two sides of the cranium.

M. Piorry's definition of ***Mania***, or ***Delirium***, is the old one of "false judgment from true premises," or "correct conclusion from false premises:" as to its seat, he observes, "it may be symptomatic of a great number of conditions of the brain; but no lesion has been discovered invariably in connexion with it, and, in very many cases, nothing has been found in the dead body to account for the phenomena observed during life." Upon *symptomatic delirium* he is more diffuse in his comments, and attributes typhoid delirium either to the disturbance of the respiration and circulation by the pressure of the tympanitic bowels upon the diaphragm and great vessels; or to the absorption and circulation through the brain of altered and diseased fluids. Here, again, he cannot admit the operation of causes of disease that are inappreciable by the senses: but, not to enter upon a discussion on this *vexata quæstio*, we dismiss the subject with this single remark, that we cannot believe that a symptom dependent upon a lesion of the brain, as some assert, or an alteration of the fluids, can be removed by so transient a cause as a dose of opium, with or without antimony, a glass of wine, or the return of day.

In regard to the coincidence of heart-disease and apoplexy, it is stated that, of twenty cases of the latter, taken at random, eight had hypertrophy, four had asthmatic affections, and several of the others were plethoric or had concretions and ossifications of the arteries.

There are some interesting experiments, with remarks, upon the effects of keeping the head of an animal, exhausted by bleeding, raised or depressed, with some highly useful practical deductions upon their bearing both on the diagnosis and treatment of cerebral affections. The opposite states of coma from congestion, and syncope from anæmia, of the brain, were induced in the animals, according to the position in which the head was maintained.

We think the following ***Special Diagnosis of Encephalitis*** and ***Softening of the Brain*** will be acceptable to our readers, as the opinion of an author who appears to have both read and observed carefully everything connected with cerebral diseases, for the express purpose of clearing up their diagnosis; though we admit that it exhibits the little advancement hitherto attained in this branch of enquiry. The experience of every one will immediately suggest how numerous are the blanks to be filled up in order to complete an accurate picture of the varied phases, not only of these two affections, but also of meningitis; of which M. P. acknowledges it is most difficult to trace the characters, from its being inseparable from disease, if not of the whole brain, at least of the parts in contiguity with the membranes. For the sake of comparison, therefore, we give an outline of its symptoms, removed from their proper place in this chapter into juxta-position with those of the two diseases more particularly under consideration.

"Meningitis, or inflammation of the membranes of the brain, is attended with vomiting, headach, and delirium at the outset; soon succeeded by convulsions, and closed with coma, paralysis, and death. But when a patient, before the age when ossifications of the arteries are usually met with, exhibiting no symptoms of disease of the heart or arteries, is seized with a dull and deep-seated pain in one side of the head, followed by sudden jerking convulsions, like those of epilepsy; when the sensibility to pain is most exquisite, which pain is excited by the slightest touch or movement of the opposite side of the body to that side of the head that is painful; when these parts become stiff and contracted, and the pain is increased by extension of the arm; when the mind is rational but obtuse,—we must dread an inflammatory softening of the brain. Again, when an old person, or one worn out or wasted by disease, especially of the organs of respiration and circulation, whose veins are varicose, whose arteries are ossified, and scarcely beat; when to a pain in the head succeeds stiffness of the muscles, with rigidity and contraction; when the convulsions are less strong, distinct, or jerking, the sensibility of the opposite side being preserved, and intelligence still perfect, then there is probably softening of the brain from obliteration of the arteries, or from retarded circulation. This conclusion will be more certain if there be no appreciable physical cause for the symptoms, nor any fever; and will be confirmed if there be any obstacle to the venous circulation in the lungs, heart, or great vessels."

Such are the symptoms of the most striking and best-marked cases, and they present but little difficulty to forming a correct diagnosis; but M. Piorry freely confesses that there are others of an intermediate kind, where it is impossible to decide to which of the two diseases they are to be referred. This exactitude of diagnosis is, however, practically of little moment; for, the softening from want of circulation being necessarily fatal, the treatment should be such as is applicable to the more curable state, the Encephalitis.

With regard to fixing the seat of the various affections of the brain, M. Piorry has nothing new to adduce; but his own experience tends rather to confirm the opinions of Gall, Serres, Foville, &c., that the grey substance is affected in delirium; the corpora striata when the lower limbs, and the optic thalami when the upper are convulsed or paralysed; that, when there is loss of speech, we may expect disease of the anterior lobes, and of the cerebellum when there is much excitement of the generative functions: but he confesses that, on all these points, and various others respecting the localization of cerebral disease, we have nothing approaching to certainty to guide us.

Great stress is laid upon the employment of quinine, in large doses, in nervous and cerebral diseases of an intermitting character, particularly in the remittent fevers of children, to whom it may be given in clysters. Even mania, when paroxysmal, will yield to it, and also the delirium of fever, when there is enlargement of the spleen. In epilepsy, when the attacks are periodic, great success has been obtained by him from the use of quinine.

There are two very instructive examples given of accurate diagnosis,—one of obliteration of the arteries of the brain, the other of cancer of the brain with paralysis of the 7th pair; but our limits will not allow us to transcribe them. The latter disease, and tubercles of the brain or its membranes, present no characteristic symptoms, and are only to be recognized by an attentive consideration of other points connected with the age and constitution of the patient.

This whole article upon the exploration and diagnosis of the brain, and its diseases, is fully as interesting as any in the work; and, though M. P. has added but little to the amount of our knowledge on these subjects, and has made a great omission in merely naming delirium tremens in conjunction with mercurial trembling, yet he has conferred a benefit on science in pointing out the extent of our ignorance, which appears to be deplorably great, both in the physiology and pathology of the cerebral organ.

There are some interesting remarks (but too long for extraction) upon *Spinal Irritation,* and the various symptomatic and sympathetic pains to which it gives rise. We are glad to see that this subject, till lately neglected, is attracting a good deal of attention in France; and we anticipate that, with their extensive opportunities and ardour for research, the French physicians will be able to add considerable information to the little we yet possess upon these minor affections of the spinal cord. At present M. P. terms the motions excited by irritation or inflammation in paralysed limbs automatic and involuntary, and refers them to the power inherent in the spinal cord of transmitting movements, howsoever excited, to the parts supplied with nerves from itself; and adds, that these motions are not more surprising than those excited by strychnine and brucine in limbs similarly affected; which, however, he does not attempt to explain.

With regard to the *Special Diagnosis of the Inflammatory Diseases of the Spinal Cord,* M. P. states his opinion that, even with the closest attention, it is very rare that we can ascertain the nature of the lesion, or even the portion of the spine that it occupies.

M. P. divides *Neuralgias,* or *Disorders of the Nerves,* into two kinds, which he calls ascending and descending, according as they have their source in the extremities or origins of the nerves. The former class he separates into those whose influence ceases on their arrival at the nervous centres, as sciatica and other forms of nervous and muscular pains; and those in which the influence extends to the corresponding sentient portion of the brain or spinal cord, and from thence is continued to the motory part, so as to produce convulsions and spasmodic motions, that vary in respect to the part of the brain affected or to the character of the neuralgia. Under this head are included epilepsy, hysteria, tetanus, and hydrophobia. In other cases a plexus forms the nervous centre, and then the affection is transmitted along the nerves that pass through it in various directions, as occurs in the pain of angina pectoris, which is felt in the arms, and that round the pelvis and down the thighs when the uterus is diseased. Descending neuralgias are instanced by the cramps, pains, and convulsions attending diseases of the brain, or when a tumour forms upon the nerve of the part affected.

M. P. describes angina pectoris under the name of Brachiothoracic Neuralgia, which he localizes in the brachial plexus and its branches, attributing the pain and palpitation of the heart to its striking against the exquisitely tender side; but he does not attempt to explain its being confined to the left side, nor why an attack should be excited by such causes as accelerate the motion of the heart. Respiration, he says, is troubled as soon as the neuralgia extends to the respiratory nerves. He has not discovered, on dissection, any ossification of the coronary arteries, nor any other organic affection of the heart, to which the disease could constantly be referred.

In his theory of *Lumbago,* as in other cases, we think M. P. has fallen into an error rather common to his countrymen, of generalizing from a few facts. In this disease, having observed two instances of lumbar pains in which ecchymosis and separation of the muscular fibres were found after death, and having also noticed that the first attacks of pain have come on after a violent blow or strain, he forthwith concludes that lumbago, pleurodynia, and almost every other muscular pain, arises from the same cause. He even adduces this theory to account for psoas abscess, which he terms an inflammation of the psoas muscle, brought on by rupture of its fibres, and extending to the surrounding cellular tissue, where it forms an abscess that finally affects the bone with caries. We do not deny that this disease may originate occasionally in this way; but we think its marked connexion with a strumous constitution ought not to have been passed over in silence.

M. P. divides *Rheumatism* into two forms,—Hæmoarthritis and Arthrohæmitis, according as the local disease precedes or follows the appearance of the general fever or inflammation of the blood; but, although he states this division to be highly practical, he neglects entirely to point out its bearing upon the treatment. This account of rheumatism, as well as of gout, we think, is improperly arranged with the diseases of the bones and joints, as though these parts constituted its primary seat. It would have been better included among the diseases of the blood, and then it would have been almost in juxta-position with diseases of the heart and pericardium, that constitute its most important and formidable complications.

Having expressed our opinions pretty fully on different parts of this work, during the course of the analytical review we have attempted to give of it, we have but little to add in conclusion. It certainly possesses very considerable merit, and testifies to the talent, ingenuity, and unwearied zeal of M. Piorry, in the investigation and application of almost every fact that can throw light upon the diagnosis of disease. Its faults are, its great size, arising from its diffuseness and the prominence given to unimportant particulars, and the desultory mode in which the subjects are treated; though the evil is remedied, in some degree, by a copious index: but it contains so much that is valuable, not only to the student, but to many who have not the advantage of attending hospital practice, and yet wish to keep on a level with the progress of medical science, that we shall be very glad to see it again, in a condensed form, and in an English dress.

The work of Dr. Suckow is one of the most striking instances we have ever met with of misapplied industry. The labour expended in its composition must have been enormous; and, although it contains an infinity of facts, we believe it to be utterly useless. Such a work, perhaps, could only be written by a German: assuredly, it will never be read by any one but a German. Its plan is, to take up separately every part and function of the human body; to enumerate, in order, every possible quality, appearance, and alteration that every such part or function can present; and then to arrange, under each head, every disease, organic or functional, of which such alteration is a sign, or in which it does or may by possibility occur! It may be conceived what a farrago of morbid affections are thus crowded together in most admired disorder, and without a shadow of scientific interest or practical benefit. The same sign is set down as indicating diseases of the most various and often directly opposite kinds, heaped pell-mell one upon another, without the slightest attempt at classification; and this, paragraph after paragraph, through 300 close-printed quarto pages! The following two paragraphs, the first from the section "*on Signs from the Position of the Head,*" and the second from one of the numerous sections "*on the Tongue,*" may be taken as very mitigated instances of the general character of the book.

"*The head hanging down, or sinking inwards*—indicates inflammation, swelling, induration of the internal parts of the neck, of the external coverings, or of the vertebræ; wounds or cicatrices on the anterior part of the neck; weakness of the muscles; depressed powers; giddiness; stupefaction; sleepiness; idiocy; pressure on the brain; dazzling; cataract; bashfulness; sadness; meditation; melancholy; incipient hydrocephalus; cerebral or spinal paralysis; apoplexy; long-continued epilepsy." (p. 34.)

"*White tongue*—indicates catarrh; aphthæ; *soor* of the mouth; tobacco-smoking; ptyalism; gastricism; scrofula; rickets; defective nutrition; long confinement; insanity; induration of the abdominal viscera; diseases of the heart; peritonitis; hepatitis; splenitis; ulcerated intestine; induration of the lungs; gastric, rheumatic, catarrhal, nervous, mucous fever; hæmorrhoids; hypochondriasis; phrenitis; meningitis; inflammation of the chest; ague with gastric complication; dyspeptic phthisis." (p. 86.)

That ever a man who had studied medicine or seen diseases should imagine that a Babylonish congregation of names of this sort could by possibility be "useful and agreeable in practice," (as the author hopes in his preface,) is really most amazing to us. But we have done our duty towards our many great and respected friends in Germany, by exposing the absurdity of the attempt.

---

